# Clean Home-delivery in Rural Southern Tanzania: Barriers, Influencers, and Facilitators

**DOI:** 10.3329/jhpn.v31i1.14755

**Published:** 2013-03

**Authors:** Donat D. Shamba, Joanna Schellenberg, Suzanne C. Penfold, Irene Mashasi, Mwifadhi Mrisho, Fatuma Manzi, Tanya Marchant, Marcel Tanner, Hassan Mshinda, David Schellenberg, Zelee Hill

**Affiliations:** ^1^Ifakara Health Institute, Mikocheni, Kiko Avenue, PO Box 78373, Dar es Salaam, Tanzania;; ^2^Faculty of Infectious Diseases, London School of Hygiene & Tropical Medicine, UK;; ^3^Faculty of Infectious and Tropical Diseases, London School of Hygiene & Tropical Medicine, UK;; ^4^Faculty of Social Sciences, University of Dar es Salaam;; ^5^Ifakara Health Institute, Mikocheni, Kiko Avenue, PO Box 78373, Dar es Salaam, Tanzania;; ^6^Faculty of Public Health and Policy, London School of Hygiene & Tropical Medicine, UK;; ^7^Swiss Tropical and Public Health Institute, Basel, Switzerland;; ^8^Tanzania Commission of Science and Technology, Dar es Salaam, Tanzania;; ^9^Faculty of Infectious Diseases, London School of Hygiene & Tropical Medicine, UK;; ^10^Center for International Health and Development, Institute of Child Health, University College London, London, UK

**Keywords:** Clean delivery, Handwashing, Formative research, Newborn, Tanzania

## Abstract

The study explored the childbirth-related hygiene and newborn care practices in home-deliveries in Southern Tanzania and barriers to and facilitators of behaviour change. Eleven home-birth narratives and six focus group discussions were conducted with recently-delivering women; two focus group discussions were conducted with birth attendants. The use of clean cloth for delivery was reported as common in the birth narratives; however, respondents did not link its use to newborn's health. Handwashing and wearing of gloves by birth attendants varied and were not discussed in terms of being important for newborn's health, with few women giving reasons for this behaviour. The lack of handwashing and wearing of gloves was most commonly linked to the lack of water, gloves, and awareness. A common practice was the insertion of any family member's hands into the vagina of delivering woman to check labour progress before calling the birth attendant. The use of a new razor blade to cut the cord was near-universal; however, the cord was usually tied with a used thread due to the lack of knowledge and the low availability of clean thread. Applying something to the cord was near-universal and was considered essential for newborn's health. Three hygiene practices were identified as needing improvement: family members inserting a hand into the vagina of delivering woman before calling the birth attendant, the use of unclean thread, and putting substances on the cord. Little is known about families conducting internal checks of women in labour, and more research is needed before this behaviour is targeted in interventions. The use of clean thread as cord-tie appears acceptable and can be addressed, using the same channels and methods that were used for successfully encouraging the use of new razor blade.

## INTRODUCTION

Ninety-nine percent of neonatal deaths occur in low- and middle-income countries, with two-thirds of these occurring in Asia and Africa (1). There have been slow declines in neonatal mortality, especially in sub-Saharan Africa (2); yet, many deaths could be prevented through basic, cost-effective interventions, such as clean delivery practices, resuscitation, thermal care, immediate and exclusive breastfeeding, care-seeking, and special care for low-birthweight babies (1).

Infections account for a large proportion of newborn deaths (3) among premature or low-birthweight infants, and with those born in contaminated environments being particularly vulnerable (4). Clean delivery practices include delivering on a clean surface, the birth attendant having clean hands during delivery, cutting and tying the cord with clean instruments and thread, and dry cord-care (putting nothing on the cord) (5,6). The importance of clean delivery behaviours for reducing infections is widely accepted; however, there is little empirical evidence on the impact of individual hygiene behaviours on infection or mortality. A recent observational study in Nepal found that birth attendants’ handwashing was associated with a 25% reduction in neonatal deaths (4), illustrating the importance of further studies in this area. As over half of the babies in sub-Saharan Africa (7) are born at home where contamination levels may be high, interventions that include clean delivery may have the potential to significantly reduce infections in newborns.

The aim of this paper is to explore hygiene-related practices among those who deliver at home in Southern Tanzania and barriers to and facilitators of behaviour change. The study was conducted as part of the formative research to design the Improving Newborn Survival in Southern Tanzania (INSIST) project, which aims to develop, implement and evaluate the effectiveness and cost of a scalable strategy to improving neonatal survival. The INSIST strategy combines home-based counselling by community volunteers, with strengthening of facility-based health system.

## MATERIALS AND METHODS

### Study area

The study was conducted in Lindi Region in Southern Tanzania. The site includes plateaus, mountainous areas, low-lying plains, and coastal areas. The site has a mixture of ethnic groups, and most people speak the language of their own ethnic group and Swahili. The area is predominantly rural. The most common occupations are subsistence farming, fishing, and small-scale trading. Most people live with their extended families in mud-walled and thatched-roof houses and get water from hand-dug wells, community boreholes, natural springs, or rivers. The health system has a network of government and NGO-owned facilities that reach the village level. Although most women attend antenatal clinic at least once in pregnancy, only 39% of births occur at facilities, and no formal system exists for postnatal checks (8).

### Data collection

Data were collected between September and December 2008 from three villages that were selected to reflect the diversity of the study area in terms of ethnicity and access to health facilities and traditional birth attendants. Eleven birth narratives and six focus group discussions (FGDs) were conducted with women who delivered at home in the last two months. Two FGDs were conducted with birth attendants [any woman who assisted someone in a home-delivery in the last year, including trained and untrained Traditional Birth Attendants (TBAs) and relatives. The last TBA training was given in the 1980s]. Respondents were selected by key informants in the village to reflect the community's sociodemographic make-up. The details of data-collection methods, with specific objectives, are shown in Table 1.

**T1able 1.** Summary of the qualitative methods

Method	Description of method	Objectives
Birth narratives	Eleven women who delivered at home in the last two months were asked for an account of the delivery and newborn care practices	Understand why women deliver at home and how they prepare for the birth.
		Understand what happens during home births and after delivery and what/who influences practices
FGDs with recently-delivering women	Six FGDs conducted with women who delivered in the last two months	To establish a broad understanding of delivery and newborn care behaviours and how these can be changed or what ‘compromise’ in behaviours could be advocated
FGDs with birth attendants and women who assist during delivery	Two FGDs conducted with birth attendants (trained and untrained birth attendants and family members who assist in delivery) attendeding at least one delivery in the last year	Understand what happens during home-births and what influences hygiene practices from the birth attendant's perspective
		Understanding the above by recently-delivering women as expressed in FGDs

The sample-size for the narratives was determined using saturation sampling (9) such that respondents were interviewed until no new information was learnt. At least two FGDs with 6-12 participants were conducted for each category of respondents.

The interviews were conducted in Swahili by two social scientists. Field notes were taken during the interviews, which were converted to detailed English transcripts (fair notes) in MS Word on the same day as the interview. All FGDs and narratives were recorded to aid the writing of fair notes and for quality assurance.

### Analysis

Data were analyzed in three categories of hygiene behaviour change: (i) delivery surface, (ii) handwashing or wearing of gloves during delivery, and (iii) cord-care (cutting, tying, and caring for the cord). A fourth category was the acceptability and likelihood of hygiene-related behaviour change. The transcripts were explored through multiple readings and key analytic categories, and themes were identified through two analysis workshops. Data were coded in NVIVO software version 7 and interpreted. Quotes in this paper are given in the third person (i.e. “She said ….”) when they came from notes and the first person (i.e. “I said .…”) when they came from recordings.

### Ethics

The study received ethical approval from the institutional review boards of Ifakara Health Institute, the National Tanzania Medical Research Co-ordinating Committee, the Tanzania Commission for Science and Technology, and the London School of Hygiene & Tropical Medicine, UK. We obtained oral and written consent at the start of each interview and FGD. The confidentiality of all study participants was assured, and names of the respondents, village, or facility are identified by code only.

## RESULTS

In total, 60 women were included in the interviews and FGDs. Respondents’ characteristics are presented in Table 2. Most of the respondents had only primary education (35 of 60) or had never been to school (22 of 60), and most mothers were between 20 and 40 years of age.

**T1able 2.** Characteristics of respondents

Type of respondent	N	Age				Parity			Education level		
		<19	19-30	>30-40	>40	1	2-4	>4	None	Primary	Secondary
Birth narrative respondents	11	1	5	4	1	1	9	1	5	6	-
Birth assistants as FGD participants	12	-	-	3	9	-	-	-	7	5	-
Recently-delivering women as FGD participants	37	5	9	17	6	12	17	8	12	24	1

The results are divided into four analysis categories: delivery surface, handwashing or wearing of gloves, dry cord-care, and the acceptability and likelihood of behaviour change.

### Delivery surface

In the birth narratives, only three of the 11 women reported delivering on an unclean cloth and one on an uncovered rope-bed. Delivering on an uncovered or unclean surface was reported as common in the FGDs. The main reason for not using a clean cloth or mat to deliver on was that the birth products were perceived as unclean; this made the use of a clean cloth seemingly unnecessary (Box 1). Respondents were uncomfortable even talking about the placenta, which was referred to in Swahili as ‘the dirty’. In FGDs, women reported that they should not even see the placenta and that the attendant should discard it immediately. The delivery cloth was often thrown away with the placenta.

Other themes relating to not using a new or clean cloth were not wanting to stain a good cloth, not using a cloth at all so the blood can fall through the rope-bed and be easily cleaned, and having an impromptu delivery, which left no time to prepare the delivery surface:

The delivery surface wasn't prepared because it was an emergency. She said they didn't put anything down, even a cloth [Age 28 years, 3 children, no education (Birth narrative)].

When a clean cloth or mat was used for covering the delivery surface, reasons were rarely mentioned, and no one mentioned the importance of hygiene for the baby's health. Health worker's advice appeared to play a strong role in delivery preparation:

These days they are told at the dispensary to use clean cloth or to wash them before use [Birth attendant, FGD].

### Handwashing or wearing of gloves

In the birth narratives, five of the 11 women reported that their birth attendants washed their hands or wore gloves, or both, during delivery. Several trained birth attendants reported that they had access to free gloves from facilities:

The nurses know me, so when my stock of gloves runs out, I just go to the dispensary to get another box of gloves [Birth attendant, FGD].

Six of the 11 women reported that a family member frequently put their hands in the vagina of delivering woman to check on the progress of labour before calling the attendant:

Her mother was entering her hand [into the vagina of delivering woman], and she was doing that frequently until she told her that the baby was coming out [Age 22 years, 2 children, no education, birth narrative].

She then inserted her hand into the vagina to know where the baby was…after a few minutes, her sister-in-law came back to see how was she doing; her sister-in-law inserted her hand again and said that she could deliver anytime [Age 20 years, 2 children, no education, birth narrative].

The reported frequency of handwashing before inserting the hand into the vagina in labour and before the actual delivery varied.

The main reason for birth attendants not washing hands or wearing gloves (Box 2) was the perception that it was an emergency situation or there was a need to rush to help the mother. The lack of water, poor access to gloves, lack of awareness, and the embarrassment of asking a birth attendant to wash their hands also emerged as minor themes. Women appeared to be able to influence wearing of gloves through purchasing and providing gloves to the birth attendant:

She showed her sister the gloves, then her sister put on the gloves, then inserted her hands to find out how far the baby was in the ‘birth door’ [Age 31 years, 3 children, completed primary school (Birth narrative)].

**Box 1.: Reasons for not using a clean cloth**

Not wanting to stain a good cloth

They don't want their new or clean mats to get bloody when delivering as it will be hard to wash them again [Birth attendant, FGD]
She removed the mat from the bed and put on some ‘dirty cloths’. I asked her why she decided to put on the dirty cloth; she said because those things that come out may spoil a cloth if you put a nice cloth [Age 30 years, 4 children, completed standard 4 (Birth narrative)]

Not wanting to waste a good cloth as the cloth will be thrown away

The old unwashed *Khanga* was put on because after delivery they can throw it away as it can't be used again [Age 18 years, 1 child, no education (Birth narrative)]
Others do not want to use new cloths or washed ones, they want to use ‘dirty’ cloths so that after delivery they don't have to wash again, instead, they dump them [Birth attendant, FGD]

Not needing a clean cloth as birth is dirty anyway

They don't use clean cloths because what is coming out with the baby is ‘dirty’, so, it is easy to dump them [Birth attendant, FGD]

Not using a cloth at all so the blood and dirt can fall to the ground and be easily cleaned

Nothing is put down for the baby so those things that come out after delivery can fall down easily [through the holes in the rope-bed] [Woman, FGD]
Some birth attendants assist some mothers to deliver on the rope-bed without putting even a cloth, and it is a common thing in the community; because it is easy to clean the bed after delivery [Birth attendant, FGD]

Respondents rarely articulated reasons for handwashing or wearing of gloves. However, gloves were described as a tool for delivery and were generally considered desirable. Only one respondent explicitly talked about gloves reducing disease. Not using gloves did not appear to influence trust in birth attendants:

We trust that the woman [referring to a birth attendant] here in our village, even though she doesn't have gloves and other things for birth [Age 20, 2 children, no education (Birth narrative)].

### Dry cord-care

All women in the birth narratives reported that a new blade was used for cord-cutting; the majority of them purchased one in advance of labour. A theme across respondent groups was that a clean blade was important to protect the baby from diseases, such as HIV or tetanus. Advice from health workers during antenatal care was also important:

We do that; we are told at the dispensary to prepare a new razor blade and new thread [Women, FGD].

New cord-ties were only reported as being used by four of the birth narrative respondents. Respondents reported that the cord was usually tied with a thread taken from a *Khanga* (a brightly-coloured piece of cloth that many East African women wear) which was rarely specified to be clean. Lack of awareness of the importance of clean thread, no preparation, and poor availability were given as reasons for not using a clean thread:

Some of them use old thread or a piece of ‘dirty’ cloth or anything nearby—but that happens to those who do not prepare for delivery [Birth attendant, FGD].

The environment where we are living leads us to use anything to tie the baby's cord. It is difficult to get the new thread if it is not prepared before [Women, FGD].

People are not aware of the consequences that can be caused by tying the cord with unclean thread [Women, FGD].

**Box 2.: Reasons for not washing hands**

Emergency situation/rush to help the mother

Her aunt didn't wash her hands before helping her to deliver. She said that it was like emergency; her aunt didn't remember to wash her hands [Age 22 years, 2 children, no education (Birth narrative)]
Washing hands will waste time while the pregnant woman is in a serious condition. In that time, we think of helping the woman and the baby only; we do not think about being clean [Birth attendant, FGD]
We don't wash hands because when you are called to help a pregnant woman you will find her ready to deliver so you will not have time to wash hands [Birth attendant, FGD]

Lack of water

She said that both her mother and her aunt didn't wash their hands before assisting her because there was not water prepared in the room where she gave birth [Age 18 years, 1 child, no education (Birth narrative)]

Lack of gloves

They depend on getting gloves from mothers … there is no medical store where from people can buy gloves; if a woman does not prepare gloves they will have no choice but using their bare hands [Birth attendant, FGD]

Lack of awareness

People do not know consequences that are caused by not washing hands when helping a delivering woman [Woman, FGD]

Nine of the 11 women in the birth narratives reported putting something on the cord (Box 3). The timing of application varied: four started applying a substance on the day the baby was born, two on day 1 or two after delivery, and two when the cord fell off (data on timing was unavailable for one woman). Substances placed on the cord included breastmilk, talcum powder, oil, petroleum jelly, ash, and dirt. The main reason for putting something on the cord was to help the cord dry and fall off:

They mix ashes and cooking oil and put those on the cord because without putting those the cord may not dry [Woman, FGD].

The cord-drying and falling off was perceived as essential for the baby's health:

When the cord gets dry for them, it is a sign that the baby is healthy [Woman, FGD].

In the narratives, those who put or advised mothers to put something on the cord were grandmothers, older women, aunts and, in one case, a health worker. In the FGDs, however, health workers were reported as advocating putting nothing on the cord:

Birth attendants do not put anything on the cord of the baby.…these days nurses at the dispensary are always telling pregnant mothers not to put anything on the cord [Birth attendant, FGD].

### Acceptability and likelihood of behaviour change

Most recommended behaviours were considered acceptable and easily changed as the main barrier to behaviour change was reported as lack of knowledge. For example, respondents felt that it would be possible to increase the use of clean cloths for delivery as these are already available:

The behaviour of not putting anything on the delivery surface is not the culture, rather it is lack of awareness…. We are not putting anything on the delivery surface because of lack of awareness [Woman, FGD].

For most behaviours, the families were responsible (e.g. providing a clean cloth, a new razor blade, and cord-tie); however, handwashing and wearing of gloves were the domain of the TBA. The TBAs reported that they would accept handwashing as soap and water were readily available. Women participants in FGDs expressed concerns about asking people to wash their hands as it would appear rude:

When you tell them to wash their hands first, they think you consider them as dirty people, and they leave you alone without any help [Woman, FGD].

The above was looked at as embarrassment of asking someone to wash their hands:

Telling them to wash their hands is just like abusing them; they won't assist you [Woman, FGD].

However, most felt that the behaviour would be adopted over time and if the community is involved. It was thought that families would also be able to prepare and buy clean threads as similar preparations were already made for razor blades, with many families saving money in pregnancy to prepare items for the delivery:

**Box 3.: Reasons for cord-care**

Reasons for how cord was cared for

When the cord gets dry for them, it is a sign that the baby is healthy [Woman, FGD]
They mix ashes and cooking oil and put on the cord because without putting that the cord may not dry [Woman, FGD]
Many people do not put anything on the cord [Woman, FGD]
They don't put anything on the cord rather than covering the cord to avoid the cord being injured [Woman, FGD]
When the cord fell off, they put breastmilk on the cord to make it dry [Age 40 years, 2 children, no education (Birth narrative)]
Also nothing was put on the cord because she was afraid of contaminating the baby; she was told at the dispensary not to put anything on the cord to make it dry [Age 31 years, 3 children, completed standard 7 (Birth narrative)].

We will prepare the thread too, like the way they prepare the new razor blade [Woman, FGD].

Dry cord-care was viewed as a less acceptable behaviour as this would be considered unhealthy for the baby:

Other people may ask why they should stop putting those things while they want to help the baby [Woman, FGD].

When asked about who should be involved in interventions around clean delivery, participants listed birth attendants, female relatives, health workers and, to help buy the new cloths and cord-ties, the husbands. Husbands are often to provide finances for women to purchase items, such as razor blades but are less involved in decision-making on birthing and pregnancy practices. The mother herself and, in the case of the first-time mothers, grandmothers and other relatives influenced the behaviours. Most mothers who delivered on unclean surface were uneducated.

## DISCUSSION

This study has identified barriers to and influencers and facilitators of clean delivery practices in rural Tanzania. In general, women and birth attendants had little knowledge of the importance of a clean delivery surface or of the importance of handwashing or wearing of gloves. Knowledge does not appear to be essential for practice because despite low knowledge, delivering on a clean cloth was the norm as reported in the narratives; quantitative data from the study area show that women frequently prepared for delivery by purchasing soap, preparing a clean cloth for drying the child, and cleaning the floor (10). The use of a new or washed cloth was also found to be common in Ghana, Uganda, and Pemba Island (11,12,13,14). This suggests that messages about preparing and delivering on a clean surface do not always need to be a focus of newborn care interventions.

Where women did deliver on an unclean surface, this was often linked to beliefs about the placenta being unclean; this belief has been reported in Asian settings (15,16,17,18), although a study in Ghana found no belief of the birth being polluting (11).

Quantitative data suggest that levels of handwashing with soap or wearing of gloves were high in this setting (46% and 58% respectively in home-deliveries) (10). Many women are still at risk of infection as family members were reported as frequently inserting their hands into the pregnant women's birth canal to check labour progress before calling the birth attendant. This could be an important source of infection and those designing interventions to promote delivery attendants having clean hands during delivery may need to focus on more than just the birth attendants. There are few data on these ‘checks’ from other countries, and this should be an area of exploration in future studies.

Most women reported that they did not prepare a clean thread for tying the cord and, in most cases, birth attendants used pieces of old cloth. This is corroborated in quantitative data from the study area, which found that only 49% of those delivering at home used a clean thread (10). It is likely that the use-rate of a clean thread could be substantially increased, using the same channels that have promoted new blade-use which is extremely high (96%) (10). The high use-rate of a new blade has been reported in other African settings (12,14). However, studies from Ghana and Uganda (11,13) found that the use-rate of a clean thread to tie the cord was also high. The variability in the use of a clean cord-tie across African settings suggest that those designing interventions need to understand the local context to aid in the selection of key behaviours that the interventions should focus on.

Behaviour change was reported as acceptable for all behaviours, except cord-care, for which there were strong beliefs about the importance of putting something on the cord to help it dry and fall off. This is similar to the findings from Ghana where researchers have suggested that promoting Chlorhexidine antiseptic application to the cord, may offer an acceptable and beneficial alternative to current practices (11).

### Strengths and limitations

Data were collected by a male and a female social scientist who are native Tanzanians and fluent in Swahili and English languages. Although Swahili was not the mother tongue of all the respondents, it is widely spoken. This enabled the researchers to understand the cultural references and aided translation of these concepts into English. As most of the respondents were women, it is possible that they were not comfortable explaining their birthing experiences in front of a male researcher. However, no differences in the details or themes were observed by the interviewers.

Different respondent groups and data-collection methods were used for gaining the views and experiences of various groups and validating the findings. It is possible that the few respondents and villages included in this study are not typical of the whole area.

### Conclusions

This study demonstrates that some good hygiene practices are already in place, despite the lack of knowledge about their importance. It also identifies several practices that need improvement: insertion of relatives’ hands into the vagina of delivering woman during labour before calling the birth attendant, the use of an unclean thread to tie the cord, and putting substances on the cord. The insertion of relatives’ hands into the vagina was an unanticipated risky behaviour that needs to be addressed in future studies which should explore how common this behaviour is in this and other setting(s). The use of a clean cord-tie appears to be acceptable, and it may be possible to improve their use in the same way that the use of clean blades for cutting the cord has been promoted. Practices for dry cord-care appears to be unacceptable, and a compromise in behaviour, such as the application of Chlorhexidine, may be needed.

## ACKNOWLEDGEMENTS

The work was supported by a grant from the Bill & Melinda Gates Foundation through Save the Children-USA (www.savethechildren.org/programs/health/saving-newborn-lives/), clinical trial number NCT00152204. We thank the participants from Lindi region. We appreciate the support of Ifakara Health Institute, particularly Shekha Nasser, Adeline Herman, Stella Magambo (RIP), Evaristus Nyanda, and Peter Madokola. Support of the Director-General of the National Institute for Medical Research-Tanzania (NIMR) is also gratefully acknowledged. INSIST was conducted as part of the African Newborn Network.
